# A Case of Shunt Vessel Closure for Symptomatic Congenital Portosystemic Shunt with Simultaneous Pancreatoduodenectomy

**DOI:** 10.70352/scrj.cr.25-0126

**Published:** 2025-05-08

**Authors:** Ayano Kakimoto, Soichi Narumoto, Naoyuki Hanari, Hisashi Gunji, Toshiharu Hanaoka, Naoto Sawada, Hisashi Mamiya, Yasushi Okazaki

**Affiliations:** Department of Surgery, Yokohama Rosai Hospital, Yokohama, Kanagawa, Japan

**Keywords:** congenital portosystemic shunt, shunt closure, pancreatoduodenectomy, hepatic encephalopathy, portal pressure, portal hypertension

## Abstract

**INTRODUCTION:**

Congenital portosystemic shunt (CPSS) is a condition in which portal blood flow bypasses the liver and directly enters the systemic circulation. CPSS is often diagnosed during childhood, but it can also be incidentally diagnosed in adulthood during imaging for other conditions. Reports of surgical treatment of CPSS in adult patients are rare.

**CASE PRESENTATION:**

A 60-year-old woman was referred to our department for further evaluation of a dilated pancreatic duct and diagnosed with ampullary carcinoma. She had a tendency toward somnolence and had taken sodium valproate for multiple seizures for 10 years. Serum ammonia level was elevated and contrast-enhanced computed tomography revealed a shunt vessel between P7 of the intrahepatic portal vein and the right hepatic vein (RHV). She was diagnosed with CPSS. Angiography showed moderate development of the intrahepatic portal system and an acceptable portal pressure increase during shunt clamping, which allows the shunt to be resected. Imaging also revealed multiple hepatic nodules with irregular shapes which was considered as focal nodular hyperplasia. Pancreatoduodenectomy (PD) with shunt closure and hepatic mass biopsy was performed. The shunt was located between P7 and RHV. The shunt was clamped at first. PD was performed while the shunt was clamped. After removing specimen, the portal pressure was 9 mmHg: this was within the acceptable range to resect the shunt. No evidence of intestinal congestion was observed, therefore the shunt vessel was closed using an automatic suturing device. On the first postoperative day, the serum ammonia level normalized. Six months after-surgery, she remained under outpatient clinic observation with no cancer recurrence. The preoperative tendency toward somnolence significantly improved.

**CONCLUSIONS:**

We report a case of symptomatic CPSS coexisting with duodenal ampullary carcinoma. The shunt closure with simultaneous PD was feasible in this case. CPSS is recommended to treat even in adult cases because it is potentially symptomatic.

## Abbreviations

CA19-9
: carbohydrate antigen 19-9
CEA
: carcinoembryonic antigen
CECT
: contrast-enhanced computed tomography
CPSS
: congenital portosystemic shunt
FNH
: focal nodular hyperplasia
HCC
: hepatocellular carcinoma
PD
: pancreatoduodenectomy
RHV
: right hepatic vein

## INTRODUCTION

CPSS is a condition in which portal blood flow bypasses the liver and directly enters the systemic circulation. CPSS is often diagnosed during neonatal mass screening or in infancy due to shunt complications. However, it can also be incidentally diagnosed in adulthood during imaging for other conditions.^1)^ Reports of surgical treatment of CPSS in adult patients are rare.^2)^ In this report, we present a case of CPSS that was identified during a detailed examination of ampullary carcinoma of the duodenum. The patient underwent a single-stage shunt vessel closure and PD. We report details of the case and provide a discussion of relevant literature.

## CASE PRESENTATION

A 60-year-old woman was referred to our department for further evaluation of a dilated pancreatic duct which was revealed by an abdominal ultrasound during a health checkup.

The patient presented in a wheelchair. Her family reported that she often appeared dazed.

Serum CEA level was 1.5 ng/mL (normal range, <5.0 ng/mL) and CA19-9 level was 19.1 U/mL (normal range, <37.0 U/mL). Serum ammonia level was elevated at 118 μg/dL (normal range, <66 μg/dL).

She had undergone surgical treatment for cerebral hemorrhage 15 years earlier and had residual left-sided hemiplegia. She had been taking sodium valproate for multiple seizures for 10 years. Although her serum ammonia level was elevated, she was not diagnosed with hepatic encephalopathy because her tendency toward somnolence was considered symptomatic due to cerebral hemorrhage. She had no history of abdominal surgery.

**Fig. 1:**
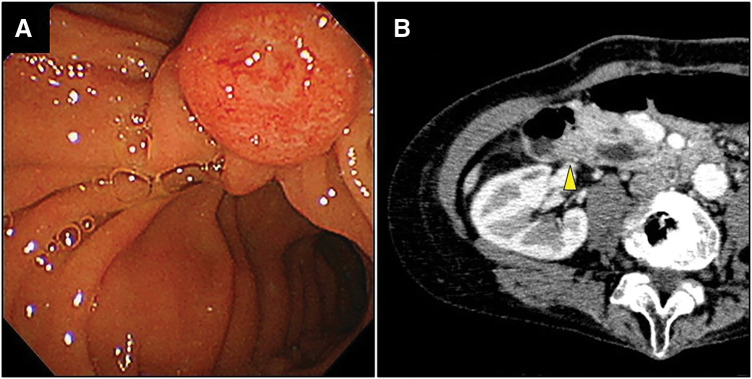
Preoperative findings. (**A**) Upper gastrointestinal endoscopy revealed an enlarged duodenal main papilla. (**B**) CECT revealed a 13-mm contrast-enhanced nodule in the duodenal papillary region (a yellow arrowhead). CECT, contrast-enhanced computed tomography

Upper gastrointestinal endoscopy revealed an enlarged duodenal main papilla, and a tissue biopsy confirmed tubular adenocarcinoma (**Fig. 1A**).

**Fig. 2:**
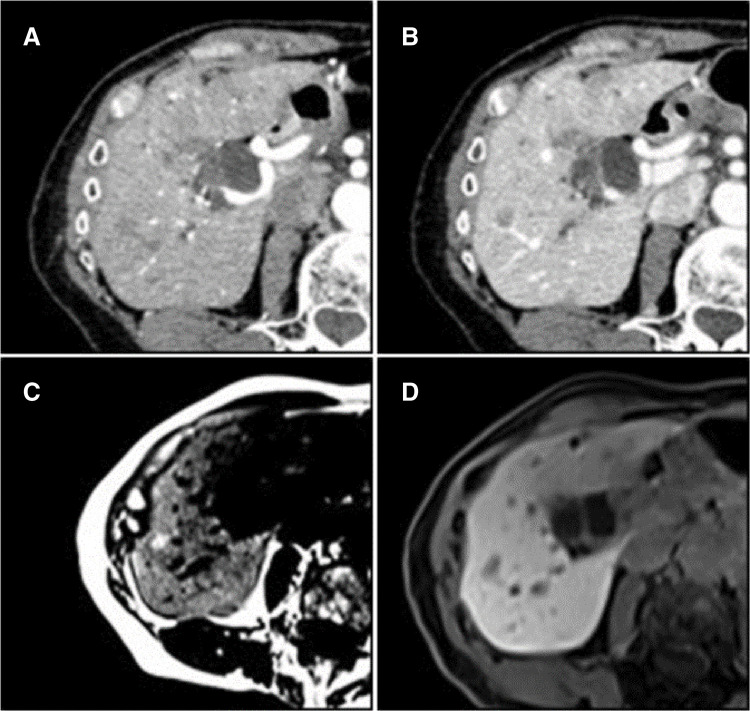
Multiple hepatic nodules. (**A**) Early phase of CECT. (**B**) Portal phase of CECT. The multiple nodules had irregular contours and were hypovascular. (**C**) The nodules showed high signal on MRI T1 weighted images. (**D**) The nodules showed mildly decreasing uptake in the hepatobiliary phase of contrast-enhanced MRI. CECT, contrast-enhanced computed tomography

CECT of the abdomen showed dilation of the common bile duct and main pancreatic duct, along with a 13-mm contrast-enhanced nodule in the duodenal papillary region (**Fig. 1B**). There was no involvement of the portal or inferior vena cava systems, and no regional lymph node enlargement was observed. A shunt between P7 of the intrahepatic portal vein and the RHVwas identified. Multiple irregular hypovascular nodules, 1 cm in diameter were observed in the liver (**Fig. 2A**, **2B**).

Abdominal contrast-enhanced MRI revealed multiple hepatic nodules with irregular shapes and decreased signal intensity in the opposed phase of T1, which indicates lipid deposits, with no early enhancement or late phase washout; a typical sign of HCC, with mildly decreased uptake in the hepatocellular phase. That suggested FNH as the primary diagnosis (**Fig. 2C**, **2D**). The metastasis and HCC were considered negative on these MRI images.

**Fig. 3:**
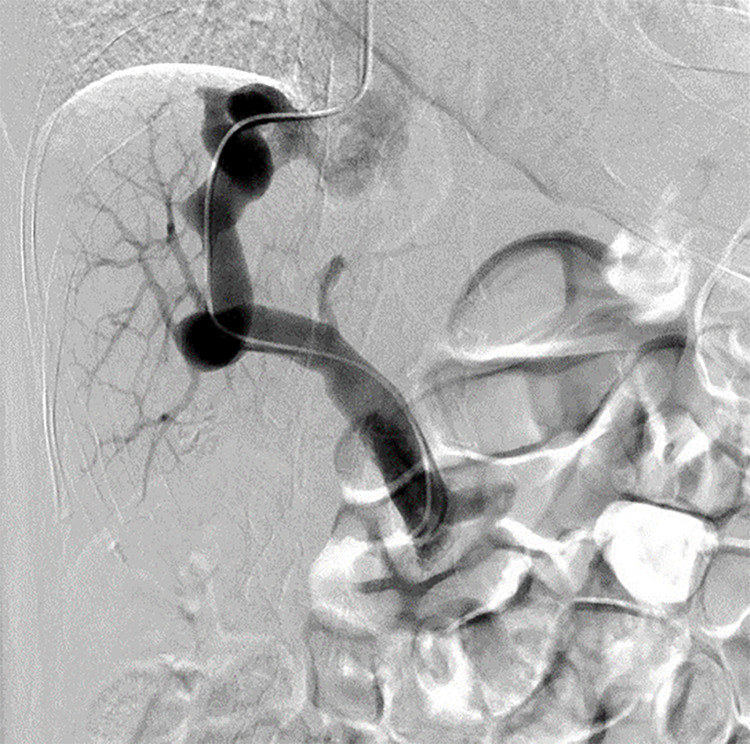
Angiography. The shunt vessel diameter was 13 mm. The intrahepatic portal vein was generally well delineated but had a narrowed and smoke-like appearance in the peripheral and left liver regions.

Angiography revealed shunt blood flow from the portal vein to the RHV, with a shunt vessel diameter of 13 mm. The intrahepatic portal vein was generally well delineated but had a narrowed and smoke-like appearance in the peripheral and left liver regions, indicating moderate development of the intrahepatic portal system (**Fig. 3**). Balloon occlusion test of the shunt vessel was performed, and the portal pressure was measured. The portal pressure was 3 mmHg before occlusion of the shunt vessel and 8 mmHg after occlusion. The shunt vessel was deemed detachable because the intrahepatic portal system was of moderate development, and shunt clamping did not significantly increase the portal vein pressure.

The preoperative diagnosis was ampullary carcinoma of the duodenum (cT3N0M0 cStage IIA according to the 7th edition of General rules for surgical and pathological studies on Cancer of the biliary tract), intrahepatic portosystemic shunt, and multiple hepatic nodules. The multiple liver nodules were diagnosed as FNH, and a biopsy was planned to confirm the diagnosis. The patient was scheduled for PD with shunt closure and hepatic mass biopsy.

**Fig. 4:**
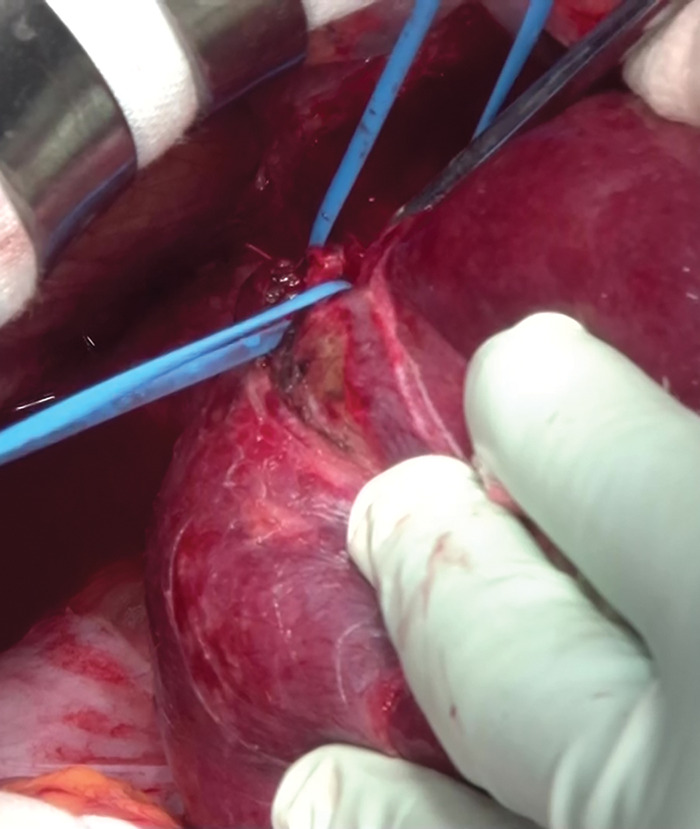
Operative finding. RHV was separated from the liver parenchyma with meticulous dissection, and the shunt was taped. RHV, right hepatic vein

Laparotomy with an upper midline abdominal incision was made. The shunt was located between P7 and RHV in the liver. RHV was separated from the liver parenchyma with meticulous dissection, and the shunt was taped (**Fig. 4**). After clamping, atrophy of the left liver lobe improved simultaneously. We punctured the portal vein with a 23G injection needle and connected it to a pressure transducer to measure the portal pressure. The pre-clamping portal pressure was 3 mmHg. PD was performed while the shunt had been clamped. After removing the specimen, the portal pressure was 9 mmHg: this was an acceptable pressure increase that allows the shunt to be resected. No evidence of intestinal congestion was observed, and shunt closure was deemed feasible. The shunt vessel was closed at its confluence with RHV using an automatic suturing device. A hyper-echoic nodule in hepatic segment S5 was identified, and mass was enucleated. Reconstruction was completed using the modified Child method. The operation lasted 6 hours and 41 minutes. The intraoperative blood loss was 644 g. No blood transfusions were required.

**Fig. 5:**
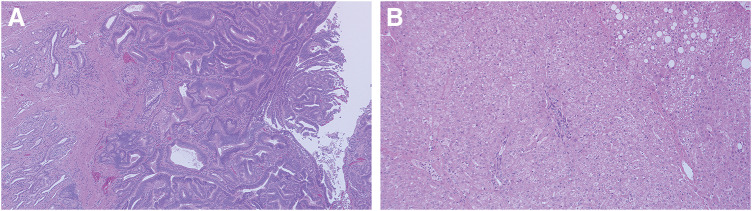
Pathological findings. (**A**) Duodenal ampullary carcinoma. (**B**) The hepatic nodules exhibited hyperplastic structures of hepatic cell cord without cytological atypia, consistent with FNH. FNH, focal nodular hyperplasia

Histopathological examination revealed duodenal ampullary carcinoma (Acd, mass type, 13 × 8 mm, well-differentiated, pT2, pPV0, pA0, INFa, Ly0, V0, Pn0, pN0, pHM0, pPM0, pEM0, pR0, pStage IB) (**Fig. 5A**). The hepatic nodules exhibited hyperplastic structures of hepatic cell cord without cytological atypia, consistent with FNH (**Fig. 5B**).

On the first postoperative day, the serum ammonia level normalized to 65 μg/dL. She was transferred to a rehabilitation hospital on the 27th postoperative day after treatment of chyle ascites, which resolved spontaneously. She was discharged on the 61st postoperative day. Six months after surgery, she remained under outpatient clinic observation with no cancer recurrence. There was no decrease in liver function pre- and postoperatively. There was a decrease in serum ammonia levels (**Table 1**). The preoperative tendency toward somnolence significantly improved, and her daytime alertness also improved. These results suggest that she had subclinical hepatic encephalopathy due to CPSS.

**Table 1:** Blood test data. There was no decrease in liver function pre- and postoperatively. There was a decrease in serum ammonia levels

	Preoperative	The third postoperative day	Six months after-surgery
AST (U/L)	19	27	17
ALT (U/L)	8	31	12
T-Bil (mg/dL)	0.41	0.3	0.44
Alb (g/dL)	3.5	2.1	4.2
NH_3_ (μg/dL)	118	58	28

Alb, albumin; ALT, alanine aminotransferase; AST, aspartate aminotransferase; T-Bil, total bilirubin

## DISCUSSION

CPSS is a congenital disorder caused by anomalies in the development of the yolk sac veins around the duodenum, as well as the persistence of yolk sac and umbilical veins, which normally regress during fetal development.^3)^ First described by Abernethy in 1793 and referred to as Abernethy malformation,^4)^ CPSS now encompasses intrahepatic portal venous shunts, extrahepatic portal venous shunts, and congenital portal vein defects. Complications of CPSS include hepatic nodular lesions due to abnormal intrahepatic portal venous flow, hepatic encephalopathy due to elevated blood ammonia levels, and pulmonary hypertension caused by microthrombi and vasoconstrictive substances derived from the shunt.^1)^

CPSS is most frequently diagnosed in children under 16 years of age.^1)^ Due to the severity of shunt complications, shunt closure is considered as a therapeutic option, either through interventional radiology or surgical closure.^1)^ In adults, CPSS is less likely to cause severe complications and is often incidentally noted on imaging studies. Reports of surgical closure of shunt vessels in adult patients are rare.^5)^ In our case, CPSS was initially detected incidentally during imaging for papillary carcinoma of the duodenum. She had never been diagnosed with hepatic encephalopathy, despite having the tendency toward somnolence. Improved consciousness and normalized hyperammonemia after the surgery suggested that her CPSS was symptomatic.

To select the treatment methods, Kanazawa et al. classified intrahepatic CPSS into 3 types based on the degree of intrahepatic portal vein development observed on angiography^6)^: mild (well-defined intrahepatic portal vein to the hepatic periphery), severe (almost completely obscured intrahepatic portal vein with smoke-like contrast), and moderate (intermediate between mild and severe). In this case, preoperative angiography showed generally good visualization of the intrahepatic portal vein, but a narrowing and smoke-like appearance in the peripheral and left hepatic regions led to a diagnosis of the moderate type.

Portal hypertension after shunt closure can be predicted by measuring the portal pressure during shunt occlusion in angiography. Shunt closure is considered to be difficult if the portal vein pressure exceeds 25–30 mmHg during occlusion.^6,7)^ In the severe type, elevated portal vein pressure at the time of shunt closure and complications such as recurrence or sepsis may occur, necessitating alternative treatment such as liver transplantation.^6)^ In this case, portal pressure was 3 mmHg before occlusion and 8 mmHg after occlusion. Increase of the portal pressure within the acceptable range without intestinal congestion indicated that shunt closure was feasible. If the portal vein pressure had elevated in angiography, we would have decided not to perform shunt closure.

Treatment of CPSS involves shunt closure or liver transplantation.^1)^ Primary shunt closure can be achieved through either endovascular or surgical approaches. Endovascular treatment involves shunt embolization with plugs or coils. Endovascular treatment is less invasive because the shunt can be embolized by puncture without operation. However, it is not recommended for patients with short or large shunts due to concerns about incomplete shunt closure and pulmonary embolization from thrombosis or the migration of embolic materials.^1,8)^ On the contrary, surgical treatment can ensure shunt closure and poses minimal risk of pulmonary embolism. In this case, the shunt was large and flowed into the proximal part of RHV, posing a high risk of pulmonary embolization; thus, surgical treatment was selected. The proximity of the shunt inflow to RHV made surgical closure advantageous, as the shunt could be taped without hepatic resection.

Shunt closure combined with other surgical procedures is rarely reported. Yanagisawa et al.^5)^ described a case of CPSS between the left gastric vein and the left renal vein, closed with simultaneous distal gastrectomy for gastric cancer. In that report, they performed distal gastrectomy, leaving only the shunt vessel. After that, a shunt clamp test was performed. This is a tip to avoid potential buffering of the portal pressure increase through the gastric marginal vein and other tissues to be resected. In our case, the shunt clamping was performed at the beginning of the procedure. PD was then performed while keeping the shunt clamped, and the specimen was removed. Approximately 2 hours and 30 minutes after clamping, the portal vein pressure was measured again and increased to 9 mmHg. Because the patient had an intrahepatic portal shunt, buffering of the portal pressure through resected tissue was unlikely, allowing for a shunt clamp test at the beginning of the surgery. If intestinal congestion due to the portal hypertension was observed after the shunt clamping, the planned shunt closure would have been to be canceled. As a strategy for a final decision to close the shunt and to reconstruct using the intestine, the safety of the surgery was ensured by a prolonged clamping time and confirming that there was no intestinal congestion.

## CONCLUSIONS

We report a case of symptomatic CPSS coexisting with duodenal ampullary carcinoma. The shunt closure with simultaneous PD was feasible in this case. CPSS is recommended to treat even in adult cases because it is potentially symptomatic.

## ACKNOWLEDGMENTS

The authors thank Dr. Naoki Hasegawa and Dr. Yukio Kakuta for making the pathological diagnosis.

## DECLARATIONS

### Funding

None.

### Authors’ contributions

AK and SN conceived of the presented idea, and analyzed and interpreted the patient’s data.

SN performed the surgery.

SN, NH, HG, TH, NS, and YO contributed to the diagnosis and treatment of the patient.

AK and SN were major contributors in writing the manuscript with support from NH, HG, TH, NS, HM, and YO.

All authors read and approved the final manuscript.

### Availability of data and materials

Not applicable.

### Ethical approval and consent to participate

This work does not require ethical considerations or approval. Informed consent to participate in this study was obtained from the patient.

### Consent for publication

The patient and her family provided informed consent to publish this case report.

### Competing interests

The authors declare that they have no competing interests.
